# *KRAS* Mutations in Squamous Cell Carcinomas of the Lung

**DOI:** 10.3389/fonc.2021.788084

**Published:** 2021-12-15

**Authors:** Fabian Acker, Jan Stratmann, Lukas Aspacher, Ngoc Thien Thu Nguyen, Sebastian Wagner, Hubert Serve, Peter J. Wild, Martin Sebastian

**Affiliations:** ^1^ Medizinische Klinik II, University Hospital Frankfurt, Frankfurt, Germany; ^2^ Dr. Senckenberg Institute of Pathology, University Hospital Frankfurt, Frankfurt, Germany; ^3^ Wildlab, University Hospital MVZ GmbH, Frankfurt, Germany; ^4^ Frankfurt Institute for Advanced Studies (FIAS), Frankfurt, Germany

**Keywords:** lung squamous cell carcinoma (LUSC), NSCLC, *RAS*, *KRAS*, review

## Abstract

*KRAS* is one of the most commonly mutated oncogenes in cancer, enabling tumor proliferation and maintenance. After various approaches to target *KRAS* have failed over the past decades, the first specific inhibitor of the p.G12C mutation of *KRAS* was recently approved by the FDA after showing promising results in adenocarcinomas of the lung and other solid tumors. Lung cancer, the most common cancer worldwide, is a promising use case for these new therapies, as adenocarcinomas in particular frequently harbor *KRAS* mutations. However, in squamous cell carcinoma (SCC) of the lung, *KRAS* mutations are rare and their impact on clinical outcome is poorly understood. In this review, we discuss the current knowledge on the prevalence and prognostic and predictive significance of *KRAS* mutations in the context of SCC.

## Introduction

Lung cancer is the most common cancer worldwide with a high mortality rate (1). Histologically, ~85% of all cases are non-small cell lung cancer (NSCLC), which is subdivided into adenocarcinoma (ADC) and squamous cell carcinoma (SCC), among others, with the latter accounting for ~30% of all NSCLC (2).

In recent decades, the development of immune checkpoint inhibitors (ICI) and agents targeting specific oncogenic driver mutations (*EGFR*, *ALK*, *ROS*, *BRAF*, *MET*, *RET*, *ERBB2* and *NTRK*) has fundamentally changed the therapeutic landscape of NSCLC by prolonging survival rates and reducing toxicities associated with conventional chemotherapy (3). While these driver mutations are common in ADC, they are rare in SCC. Instead, the mutational landscape of SCC consists of genes such as *TP53*, *CDKN2A*, *PTEN*, *PIK3CA*, *KEAP1*, *MLL2*, *HLA-A*, *NFE2L2*, *NOTCH1*, *FGFR1-4*, and others (4, 5). Unfortunately, there are no approved therapies that target any of these mutations, so chemotherapy and ICI are usually the only therapeutic approaches for advanced-stage SCC patients (6).

The most common driver mutation in NSCLC patients is found in the *Kirsten rat sarcoma virus oncogene homolog* (*KRAS*) gene. *KRAS* mutations are quite common in ADC with an incidence of ~25% in Western countries and ~10% in Asian populations and are associated with past and current tobacco use (7). In recent years, several studies pointed to a variety of effects of *KRAS* mutations on ADC biology, namely, patterns of metastatic spread, efficacy of chemotherapy, ICI, and *EGFR* therapy, as described elsewhere (8, 9). In addition, there are less frequently mutated isoforms of *KRAS*, namely, *NRAS* (*neuroblastoma rat sarcoma viral oncogene homolog*) and *HRAS* (*Harvey rat sarcoma viral oncogene homolog*) (10). In contrast to ADC, *RAS* mutations are rare in SCC.

Recently, after decades of unsuccessful efforts, sotorasib, a selective inhibitor of *KRAS* with codon 12 glycine to cysteine substitutions (p.G12C), has been approved by the *U.S. Food and Drug Administration* (FDA) after showing promising results in phase I and II trials in NSCLC and other solid tumors (11–13). Adagrasib, another *KRAS* p.G12C inhibitor, is currently being studied in clinical trials (14). In addition to direct inhibition, there are other approaches to target *KRAS*. BI 1701963, for example, is an inhibitor of *SOS1*, which in turn is an important mediator of *KRAS* activation as a guanine nucleotide exchange factor (15). The farnesyltransferase inhibitor tipifarnib received breakthrough therapy designation from the FDA for *HRAS*-mutated head and neck SCC (16). Furthermore, combined inhibition of multiple pathways of the *KRAS* signaling network may provide clinical benefit in patients with *KRAS* p.G12D-mutated tumors, as shown by preclinical data of the *MEK* inhibitor trametinib in combination with the *ROS1/TRK* and *SRC/FAK/JAK2* inhibitor repotrectinib (17).

Since the clinical relevance of *RAS* mutations in the context of SCC is unclear and new therapies are emerging, the current state of knowledge will be discussed here.

## Prevalence and Incidence

### Cancer Mutation Databases

Cancer mutation databases are one way to estimate the prevalence of *KRAS* mutations in SCC (**Table 1**). Probably the largest mutation database is the *Catalogue of Somatic Mutations in Cancer* (COSMIC), whose data come from over 25,000 manually curated publications totaling over 1.3 million tumor samples (30). At the time of writing, COSMIC provides over 5,000 samples of SCC patients tested for *RAS* mutations, with mutation rates of 3.5, 1.0, and 0.8% for *KRAS*, *NRAS*, and *HRAS*, respectively. Among patients with *KRAS* mutations, the most common amino acid substitutions were p.G12D (28%), p.G12C (27%), and p.G12V (16%).

**Table 1:** Studies outlining the prevalence of *KRAS* mutations in SCC.

Study/author	Study type	Country/region	SCC patients tested	Prevalence of *KRAS* mutations	Comment
TCGA (18)	Pan cancer mutation database	USA	n = 841	1.3%	
MSKCC, 2017 (19)	Pan cancer mutation database	USA	n = 170	6.5%	
ETOP Lungscape, 2018 (4)	Multi-center, retrospective	Europe	n = 888, stage I-IIIA	6.1% (54 cases, p.G12C 50%, p.G12D 11.5%, pG12A 9.6%, p.G13C 9.6%)	
CRISP (20)	Multi-center, prospective register study	Germany	n = 110, stage IIIB-IV	4.6% (5 cases, 1 p.G12C, 4 non-p.G12C)	
CHOICE (21)	Multi-center, retrospective	China	n = 114, all stages	0%	*HRAS*: 4 cases 96% men
Wang et al. (22)	Single-center, retrospective	China	n = 310, all stages	2.6% (8 cases)	No correlation with *PIK3CA* status was found
Fiala et al. (23)	Single-center, retrospective	Czech Republic	n = 215, all stages	7.4% (16 cases, 8 p.G12C, 1 p.G12D, 1 p.G12A, 1 p.G12V)	No correlation with sex or smoking status was found
Tao et al. (24)	Single-center, retrospective	China	n = 157, all stages	4.5% (8 cases, 5 p.G12D, 1 p.G12C, 1 p.G12V)	92.4% men
Yim et al. (25)	Single-Center	Korea	n = 104, all stages	1.9% (2 cases)	*NRAS*: 1 case
La Fleur et al. (26)	Single-center, retrospective	Sweden	n = 102, all stages	4.9% (5 cases)	*NRAS*: 1 case, *HRAS*: 2 cases
Wang et al. (27)	Single-center, retrospective	China	n = 46, all stages	4.3% (2 cases)	
Rekhtman et al. (28)	Single-Center, retrospective	USA	n = 95, all stages	0%	
Lee et al. (29)	Single-Center	Korea	n = 26, all stages	14% (4 cases)	*HRAS*: 65%

*The Cancer Genome Atlas* (TCGA) is a curated, open-access database providing comprehensive and likely refined genomic, epigenetic, transcriptomic and proteomic data of more than 11,000 patients with various cancer types (18). TCGA dataset, accessible *via* the *cBioPortal* at https://www.cbioportal.org/ (31), currently contains samples from 841 SCC patients with available mutation data. Of these 841 patients, mutations in *KRAS*, *NRAS*, and *HRAS* occurred in 1.3, 0.8, and 2.0% of cases, respectively. Interestingly, only 3 of the 11 samples with *KRAS* mutations were codon 12 mutations.

The *MSK-IMPACT Clinical Sequencing Cohort* (MSKCC), in which samples from a total of over 10,000 patients were analyzed, included over 1,500 NSCLC patients. Of all 170 SCC patients, 11 showed a *KRAS* mutation (6.5%) with the most common being p.G12C (n = 5) followed by p.G12V (n = 2) (19).

### Multi-Center Studies

**Figure 1:**
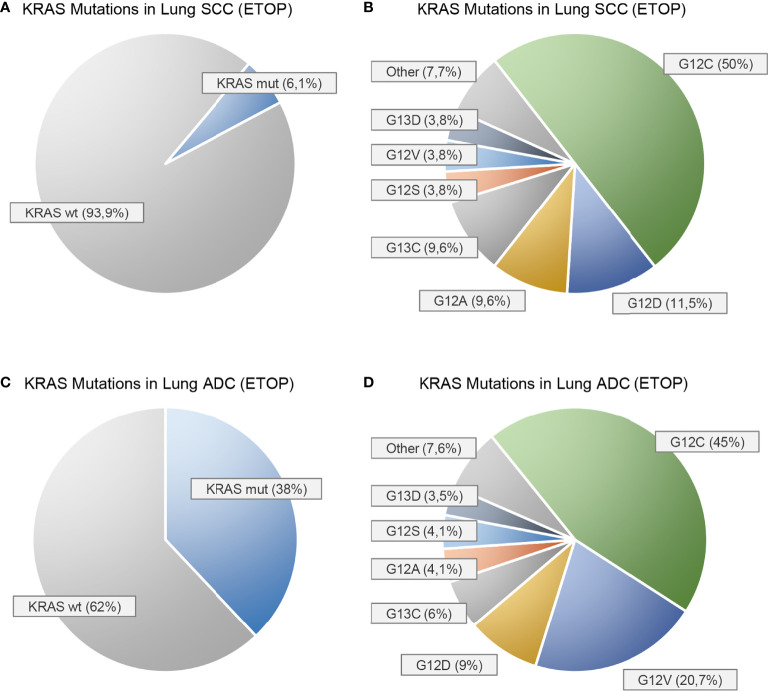
Prevalence of KRAS mutations in squamous cell carcinomas **(A, B)** and adenocarcinomas **(C, D)** of the lung as reported by the European Thoracic Oncology Platform (ETOP) Lungscape iBiobank [Ref. (4)]. **(A, C)** show the proportion of KRAS mutated (mut) and KRAS wild-type (wt) patients. **(B, D)** show the frequencies of the individual KRAS mutations.

As part of the *European Thoracic Oncology Platform’s* (ETOP) *Lungscape* project, PCR multiplex tests were performed on tumor samples of 888 patients of 17 different sites with resected stages I–IIIA SCC. In this cohort, a *KRAS* mutation was found in 6.1% (4). The most common mutations were p.G12C (50%), p.G12D (11.5%), p.G12A, and p.G13C (9.6% each) (**Figure 1**). Mutation rates did not differ significantly between current/former smokers and never-smokers (6.3% vs. 5.0%, p >0.99). However, as expected, the group of never-smokers was quite small with three mutations in 60 patients.

An analysis from the Germany-wide prospective *CRISP* registry with more than 150 participating centers reported a *KRAS* mutation rate of 4.6% in 109 SCC patients (20). In contrast, no *KRAS* mutation was detected in 114 SCC patients at 6 hospitals in China by whole-exome sequencing in the retrospective CHOICE study. However, *HRAS* mutations occurred in 3.5% of cases (21).

### Single-Center Studies

A study of 310 SCC patients from China revealed 8 cases of *KRAS* mutations (2.6%) that did not correlate with *PIK3CA* mutation status (22). In another work on 157 Chinese patients with early-stage SCC, *KRAS* mutations were detected in 4.5% of all cases (5 of 8 patients had a p.G12D mutation, 1 p.G12C, 1 p.G12V, and 1 other). Male patients were particularly prevalent in this study, accounting for 92.4% (24). Another study from China with 46 SCC patients reported a prevalence of *KRAS* mutations of 4.3% (27).

In a retrospective single-center study from the Czech Republic with a total of 215 patients with advanced SCC, a *KRAS* mutation was described in 7.4% (23). Approximately 68.8% of these mutations were in codon 12, with p.G12C being the most common mutation (50%). As in the ETOP study, no correlation with sex or smoking status was observed. In a study of 102 Swedish SCC patients, mutation rates of 4.9, 1.0, and 2.0% were described for *KRAS*, *NRAS*, and *HRAS*, respectively (26). In contrast, another retrospective study examined 95 resected SCC and found no *KRAS* mutation (28).

A retrospective study from Korea, in which whole-exome sequencing of 104 SCC samples was performed, revealed 2 *KRAS* mutations and 1 *NRAS* mutation (25). In contrast, another Korean study using only 26 SCC found 14% *KRAS* and 65% *HRAS* mutations (29). The paper gave no indication of a cause for these very high mutation rates compared with other publications.

### Estimated Incidence

Using mutation prevalence data of the COSMIC and TCGA databases and cancer incidence data from the *American Cancer Society*, Prior et al. estimated an incidence of 2,794 new *KRAS*-mutated SCC cases per year (782 for *KRAS* p.G12C) in the United States (32). This is roughly equivalent to an incidence of 0.85 per 100,000 inhabitants per year (0.23 per 100,000 inhabitants per year for *KRAS* p.G12C mutant SCC).

## Prognostic and Predictive Value

Because *KRAS* mutations are rare in SCC, there are few data on the impact of mutations on patient outcomes. Therefore, pooled analyses are a promising tool to find correlations. To our knowledge, there are two such pooled cohorts of interventional trials.

Zer et al. evaluated data from four double-blind, placebo-controlled *National Cancer Institute of Canada Clinical Trials Group* (NCIC CTG) trials of *EGFR* tyrosine kinase inhibitors (TKIs) (33). The four studies were BR.21 (erlotinib in second- and third-line stages III-IV NSCLC) (34), BR19 (adjuvant gefitinib in completely resected stages IB-IIIA NSCLC) (35), BR.26 (dacomitinib in stages IIIB-IV NSCLC after first-line chemotherapy and EGFR TKI) (36), and TOPICAL (first-line erlotinib in advanced stage NSCLC unsuitable for chemotherapy) (29). All studies enrolled patients regardless of their *EGFR* mutational status. In the retrospective analyses, *KRAS* and *EGFR* mutation status was determined for all four studies. The pooled analysis with a total of 1,362 patients included 385 SCC cases in which *KRAS* mutations were present in 6% (45% p.G12C/p.G12V; 32% p.G12D/p.G12S; and 20% p.G12A/p.G12R). Although no significant impact on overall survival (OS) was found, supplemental data showed that *KRAS* mutation tended to have a negative impact on OS (hazard ratio [HR] 1.288, 95% confidence interval [CI] 0.68–2.43) for the 177 evaluable patients.

The other pooled analysis of prospective trials was performed by Shepherd et al. (37). This study included four trials evaluating adjuvant chemotherapy versus observation in patients with resected early-stage NSCLC, including JBR.10 (vinorelbine plus cisplatin in stages IB-II resected NSCLC) (38), IALT (cisplatin-containing doublet in stages I–III resected NSCLC) (39), ANITA (vinorelbine plus cisplatin in stages IB–IIIA NSCLC) (40), and CALGB 9633 (paclitaxel plus carboplatin in stage IB resected NSCLC) (41). Of the 707 evaluable SCC patients, 6% had a *KRAS* mutation. In this analysis, OS tended to be worse in the presence of a *KRAS* mutation with a HR of 1.41 (95% CI, 0.89–2.23). No difference was observed between patients in the treatment and observation groups.

Apart from these two studies, there are mainly retrospectively collected data. Although there are plenty of studies examining the impact of *RAS* on survival in ADC, data on *RAS* mutant SCC are seldom reported. A systematic review from 2004 by Mascaux et al. comprised 4 retrospective studies reporting survival data on *RAS* mutant SCC (42). With a mutation rate of 7.1% in 280 patients, OS did not significantly differ between mutant and wild-type group with a HR of 1.49 (95% CI, 0.88–2.52). It can be assumed that most patients included in this review only received surgery and/or radio-/chemotherapy.

In a recent paper Xu et al. studied 64 Chinese patients with advanced-stage SCC who underwent ICI (43). Overall, the seven patients (10.9%) with a *KRAS* mutation had a higher overall response rate than wild-type patients (71.4% vs. 22.8%, p = 0.009). Yet, progression-free survival (PFS, HR 0.62, 95% CI 0.26–1.48) and OS (HR 0.68, 95% CI 0.2–2.3) did not differ significantly.

However, in another retrospective single-center study by Fiala et al. of 223 Czech SCC patients treated with *EGFR* TKI (*EGFR* wild-type *and* mutant), OS differed significantly between *KRAS* mutant (7.4%) and wild-type patients with a median survival of 5.7 vs. 8.2 months (p = 0.039) (23). Anyway, PFS was not significantly different between the groups. Of note, only univariate analysis was done in this study diminishing its validity.

In contrast, Tao et al. reported 157 Chinese SCC patients, 4.5% of whom had a *KRAS* mutation. OS and DFS were not significantly different between groups with a HR of 0.91 (95% CI 0.22–3.76) and 0.56 (0.14–2.28), respectively (24).

## Discussion

### Controversies Over *KRAS* Status Related to SCC

As pointed out, *KRAS* mutations in SCC are rare and appear to range from 1 to 7% in most publications (**Table 1**). It does not seem to differ significantly between Western and Eastern populations, as is the case with ADC (7).

The low prevalence has led some authors to suggest that there are no *KRAS*-mutated SCC and that reported cases may be misclassified ADC or adenosquamous carcinoma (ADSQC) (28). A phenomenon supporting this hypothesis might be ADC to SCC transition (AST). Data from genetically engineered mouse models (44, 45) show that the loss of *LKB1*/*STK11* of *KRAS* mutated ADC can lead to an ADC to SCC transition (AST) as reviewed in (46). Besides mutational events, transcriptional reprogramming was also shown to be a potential driver of AST (47). As a consequence, cases of *KRAS* mutated SCC (or ADSQC) might in part be the result of an AST with very early onset. In line with this, *KRAS* mutations do occur in both the squamous and the glandular components of ADSQC (48, 49).

In their very thorough study, Rekthman et al. reviewed all six cases of *KRAS* mutated SCC known to their department by repeating immunohistochemistry (IHC) and morphological examination (28). Two of the cases were reclassified as ADSQC after detection of glandular components in other specimens of the same tumor (preoperative biopsy vs either metastatic/recurrent site or same-site resection). Another three cases turned out to be poorly differentiated ADC, defined as ΔNp63-negativity/TTF1-positivity in IHC (50). The last case consisted of two distinct cell populations: the first one ΔNp63-positive/TTF1-negative (~5%) and the second one ΔNp63-negative/TTF1-positive (~95%). Morphologically, this tumor was predominantly squamous with ~5% glands. As this is suggestive for an ADSQC but does not match the *World Health Organization* definition of ADSQC, which requires more than 10% of each component (51), the authors classified this tumor as “biphasic tumor with <10% glands”. Overall, the authors concluded that *KRAS*-mutated SCC may in some cases represent misclassified ADSQC or poorly differentiated ADC.

Indeed, morphological differentiation between ADC and SCC can be difficult in some cases, and small sample sizes and poor differentiation are known diagnostic pitfalls (52). Therefore, it is very likely, that at least some *KRAS*-mutant SCC described in the literature are actually misclassified ADC or ADSQC. However, given the low reported frequency of *KRAS* mutations, the sample size of Rekthman et al. (28) appears too small for definitive conclusions.

Given the continuity with which *KRAS* mutations are described in the literature, it is unlikely that there are no *KRAS*-mutant SCC. Nevertheless, we believe it is reasonable to closely review cases of *KRAS*-mutated SCC in collaboration between clinicians and pathologists.

### Lack of Data and Potential Bias

In recent years, many studies have attempted to correlate outcome parameters with the presence of specific *KRAS* mutations associated with ADC, including prognostic value, predictive value (e.g., with *EGFR* TKI, ICI, etc.), patterns of metastatic spread, and other aspects, as described elsewhere (8, 9). In any case, these results cannot be simply extrapolated to SCC.

Clearly, the rarity of *KRAS* mutations in SCC complicates the ability to conduct such studies. However, in our review of the literature, even if larger cohorts were studied, we noticed a tendency to either not stratify patient outcomes with KRAS-mutated NSCLC by histology, or, at most, to mention them in the supplementary information (4, 21, 26).

Also, the fact that the College of American Pathologists (CAP), the International Association for the Study of Lung Cancer (IASLC), the Association for Molecular Pathology (AMP), and the European Society of Clinical Oncology (ESMO) do not recommend KRAS mutation testing in SCC (52, 53) may introduce bias in retrospective studies.

## Conclusion

As mentioned earlier, new drugs that directly or indirectly target mutant *KRAS* are in development or have been approved recently (8, 9). Although *KRAS* is much more commonly mutated in ADC, a small proportion of 1–7% of SCC patients may also benefit from these new therapies. However, the biological effects of *KRAS* mutations are highly dependent on the tumor cell of origin (54) and there is limited evidence in SCC. Whether the outcome of *KRAS*-directed therapy is different in ADC, SCC, and other solid tumors is yet unknown. Therefore, we welcome the fact that the ongoing studies of sotorasib and adagrasib include NSCLC with non-adenocarcinomatous histology (NCT04303780, NCT04685135). One patient with SCC has already been enrolled in the CodeBreaK100 phase II study of sotorasib (11).

Against the background that lung cancer is the most common malignancy worldwide, the incidence of *KRAS*-mutated SCC may seem low with roughly 0.85 per 100,000 inhabitants per year. Nevertheless, it represents a relevant subgroup in an increasingly segmented clinical and therapeutic landscape.

## Author Contributions

FA and MS contributed to conception and review of the literature. FA wrote the first draft of the manuscript. SW and PW wrote sections of the manuscript. All authors contributed to the article and approved the submitted version.

## Conflict of Interest

PW has received consulting fees and honoraria (private/institutional) for lectures by Bayer, Janssen-Cilag, Novartis, Roche, MSD, Astellas Pharma, Bristol-Myers Squibb, Thermo Fisher Scientific, Molecular Health, Sophia Genetics, Qiagen, Eli Lilly, Myriad, Hedera Dx, and Astra Zeneca. MS has received consulting fees and honoraria for lectures by Novartis, BMS, Roche, Lilly, Boehringer-Ingelheim, Pfizer, MSD, Astra-Zeneca, Celgene, AbbVie, Takeda, Janssen-Cilag, and Tesaro. JS has received consulting fees and honoraria for lectures by Novartis, BMS, Leo Pharma, Oncopeptides, Astra-Zeneca, Roche, Takeda, Boehringer-Ingelheim, Amgen, and Pfizer.

The remaining authors declare that the research was conducted in the absence of any commercial or financial relationships that could be construed as a potential conflict of interest. The handling editor declared a past co-authorship with one of the authors MS.

## Publisher’s Note

All claims expressed in this article are solely those of the authors and do not necessarily represent those of their affiliated organizations, or those of the publisher, the editors and the reviewers. Any product that may be evaluated in this article, or claim that may be made by its manufacturer, is not guaranteed or endorsed by the publisher.

## Abbreviations

ADC, Lung adenocarcinoma; ADSQC, Adenosquamous carcinoma; AMP, Association for Molecular Pathology; AST, Adenocarcinoma to squamous cell carcinoma transition; CAP, College of American Pathologists; CI, Confidence interval; COSMIC, Catalogue of Somatic Mutations in Cancer; EGFR, Epidermal growth factor receptor; ESMO, European Society of Clinical Oncology; ETOP, European Thoracic Oncology Platform; FDA, U.S. Food and Drug Administration; HR, Hazard ratio; HRAS, Harvey rat sarcoma viral oncogene homolog; IASLC, International Association for the Study of Lung Cancer; ICI, Immune checkpoint inhibitor; IHC, Immunohistochemistry; KRAS, Kirsten rat sarcoma viral oncogene homolog; LKB1, Liver kinase B1; MSKCC, MSK-IMPACT Clinical Sequencing Cohort; NCIC CTG, National Cancer Institute of Canada Clinical Trials Group; NRAS, Neuroblastoma rat sarcoma viral oncogene homolog; NSCLC, Non-small cell lung cancer; OS, Overall survival; p.G12C, Codon 12 glycine to cysteine substitution; PCR , Polymerase chain reaction; PFS, Progression-free survival; RAS, Rat sarcoma viral oncogene homolog; SCC, Lung squamous cell carcinoma; STK11, Serine/threonine kinase 11; TCGA, The Cancer Genome Atlas; TKI, Tyrosine kinase inhibitor.
